# Classification of bacterial plasmid and chromosome derived sequences using machine learning

**DOI:** 10.1371/journal.pone.0279280

**Published:** 2022-12-16

**Authors:** Xiaohui Zou, Marcus Nguyen, Jamie Overbeek, Bin Cao, James J. Davis

**Affiliations:** 1 Laboratory of Clinical Microbiology and Infectious Diseases, Department of Pulmonary and Critical Care Medicine, Center for Respiratory Diseases, China-Japan Friendship Hospital, National Clinical Research Centre for Respiratory Disease, Beijing, China; 2 Data Science and Learning Division, Computing Environment and Life Sciences Directorate, Argonne National Laboratory, Lemont, IL, United States of America; 3 Consortium for Advanced Science and Engineering, University of Chicago, Chicago, IL, United States of America; Tianjin University, CHINA

## Abstract

Plasmids are important genetic elements that facilitate horizonal gene transfer between bacteria and contribute to the spread of virulence and antimicrobial resistance. Most bacterial genome sequences in the public archives exist in draft form with many contigs, making it difficult to determine if a contig is of chromosomal or plasmid origin. Using a training set of contigs comprising 10,584 chromosomes and 10,654 plasmids from the PATRIC database, we evaluated several machine learning models including random forest, logistic regression, XGBoost, and a neural network for their ability to classify chromosomal and plasmid sequences using nucleotide k-mers as features. Based on the methods tested, a neural network model that used nucleotide 6-mers as features that was trained on randomly selected chromosomal and plasmid subsequences 5kb in length achieved the best performance, outperforming existing out-of-the-box methods, with an average accuracy of 89.38% ± 2.16% over a 10-fold cross validation. The model accuracy can be improved to 92.08% by using a voting strategy when classifying holdout sequences. In both plasmids and chromosomes, subsequences encoding functions involved in horizontal gene transfer—including hypothetical proteins, transporters, phage, mobile elements, and CRISPR elements—were most likely to be misclassified by the model. This study provides a straightforward approach for identifying plasmid-encoding sequences in short read assemblies without the need for sequence alignment-based tools.

## Introduction

Plasmids are extrachromosomal genetic elements that typically replicate autonomously, especially in several clinically important bacterial pathogens [1]. Plasmids often carry various genes conferring host virulence and antimicrobial resistance [2]. Bacteria can exchange genetic information using a plasmid as a “vehicle” through inter-host conjugation [3], which is one of the most effective mechanisms for driving evolution and adaptation [4]. For example, a plasmid carrying a carbapenemase gene can be transferred to a susceptible *Klebsiella pneumoniae* (KPN) isolate, rendering it resistant to carbapenems [5]. Some virulence islands carried on plasmids can enhance the pathogenicity of a recipient bacterium and accelerate adverse clinical outcomes [6]. Therefore, accurate plasmid identification is pivotal for fully understanding gene flow in diverse environments, particularly in clinical settings.

Traditionally, plasmids were isolated from bacterial cultures using alkaline lysis and then sequenced to get their genetic information. However, these methods of identification are laborious and work best on smaller plasmids. Although culture-dependent methods have traditionally been used to isolate plasmids, including Pulse Field Gel Electrophoresis (PFGE), these methods are difficult to perform in high throughput [7]. Metagenomic sequencing methods can overcome limitations of traditional methods that require culturing or plasmid isolation [8]; however, these methods often produce numerous contigs which require additional bioinformatic analyses to determine if they are from plasmids or chromosomes [9].

Many bioinformatic tools have been developed to identify plasmid sequences from the assembled genome sequences of pure isolates or metagenomic samples. Most of them are based on BLAST searches [10] against well-curated plasmid sequence databases such as PlasmidFinder or PLASDB [11–13]. PlasmidFinder is a user-friendly, web-based program that aims to identify plasmid replicons in bacterial genome sequences [11]. PLASDB is a resource containing over 34,000 plasmid records collected from the NCBI nucleotide database and provides users with an interactive view of all obtained plasmids with additional metadata such as sequence characteristics, sample-related information, and taxonomy [12]. Although these tools are highly accurate, their accuracy is scoped to the sequence diversity within their respective database. For instance, PlasmidFinder was established for *in silico* detection and typing of plasmids for members of the *Enterobacteriaceae*, and PLSDB was built on bacterial plasmids retrieved from the NCBI nucleotide database.

Another promising approach to differentiating plasmid and chromosomal contigs is the use of machine learning (ML) techniques [14]. ML models can potentially learn unique sequence features that differentiate plasmids and chromosomes, and recently, several studies that use ML models to distinguish plasmid and chromosome sequences have been published [15–18]. For example, PlasFlow employs a neural network for identifying bacterial plasmid sequences in environmental samples and achieved accuracies of up to 96%. PlasClass uses a logistic regression model that utilizes k-mer frequencies of plasmid and chromosomal subsequences with lengths up to 100kb. It achieved an accuracy of 96.9% with 100kb-length fragments. Another published tool called Mlplasmids uses 5-mer frequencies and a support-vector-machine based model to predict plasmid and chromosomal contigs from *Enterococcus faecium*, *Klebsiella pneumoniae*, and *Escherichia coli* [15].

Although the use of ML is showing considerable promise in the classification of plasmid and chromosomal sequences, more work is required to understand how the accuracy is affected by the choice of ML algorithms, sequence lengths, k-mer frequencies, and mobile genetic sequences within each element. In this study, we address these questions by building ML models and evaluating their ability to discriminate plasmids from chromosomes.

## Materials and methods

### Datasets and feature extraction

Plasmid and chromosome sequences were retrieved from the PAThosystems Resource Integration Center (PATRIC) database (now called the Bacterial and Viral Bioinformatic Resource Center, BV-BRC) [19,20]. We collected every genome labeled with a good quality score [21] with either “complete” or “WGS” assembly status from GenBank [22] that had the plasmids and chromosomes clearly labeled [19]. A training set was developed using 10,584 bacterial chromosomes with lengths greater than10kb and 10,654 plasmid genomes with lengths greater than 2kb. Additionally, taxonomic information was also downloaded for each sequence used. Contigs containing ambiguous nucleotides were omitted.

**Fig 1:**
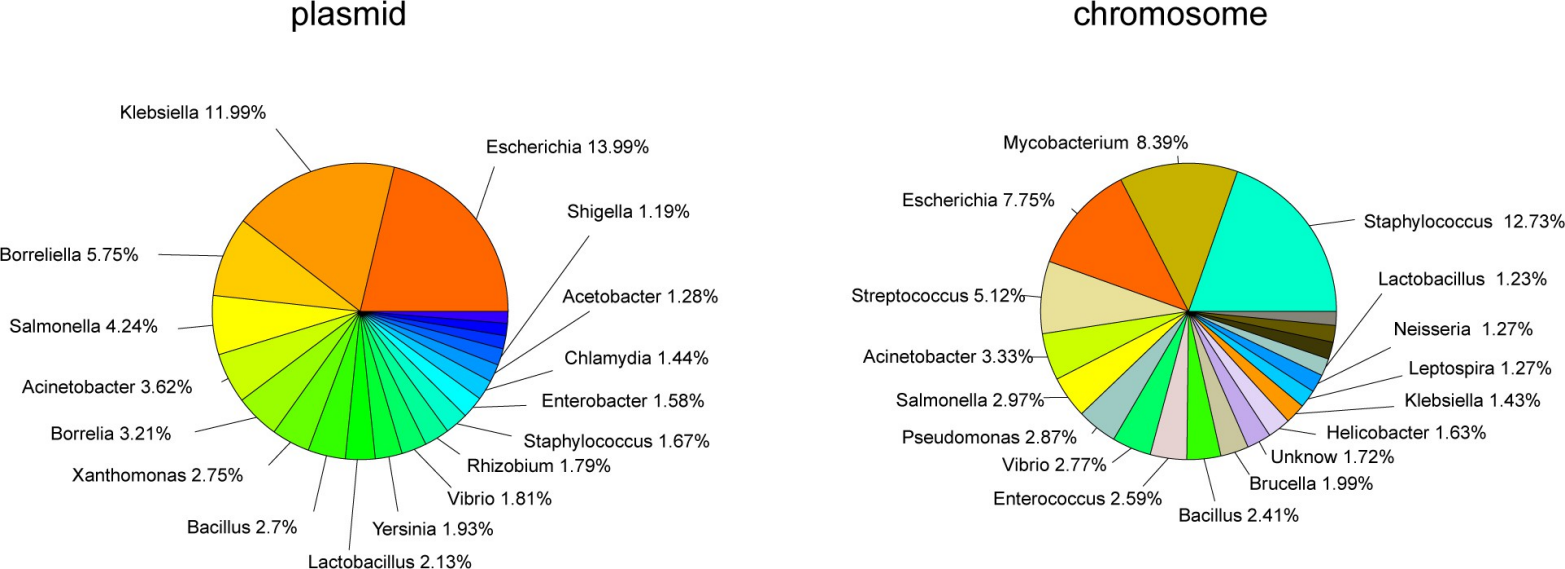
Top 20 genera in the plasmid and chromosomal datasets. A total of 10,654 chromosomal contigs and 10,584 plasmid contigs were used. Genera representing greater than 1% of the contigs are labeled.

The plasmid dataset contained a total of 1,258 species and 485 genera, and the chromosomal dataset contained 2,212 species and 906 genera. In the chromosomal set, *Staphylococcus*, *Mycobacterium*, and *Escherichia* were the top three genera, accounting for 12.7%, 8.4%, and 7.7% of the data respectively. In the plasmid set, *Escherichia*, *Klebsiella*, and *Borreliella* were the top three hosts, comprising 14.0%, 12.0% and 5.8% of the total plasmid contigs, respectively (**Fig 1**). *Escherichia*, *Acinetobacter*, *Salmonella*, *Vibrio*, *Bacillus*, *Borrelia*, *Klebsiella*, *Lactobacillus*, *Staphylococcus* existed in among the top 20 genera in both the plasmid and chromosome datasets. Prior to building models, 1,000 plasmid and 1,000 chromosomal sequences were separated from the dataset to create a holdout set.

### Model generation

**Fig 2:**
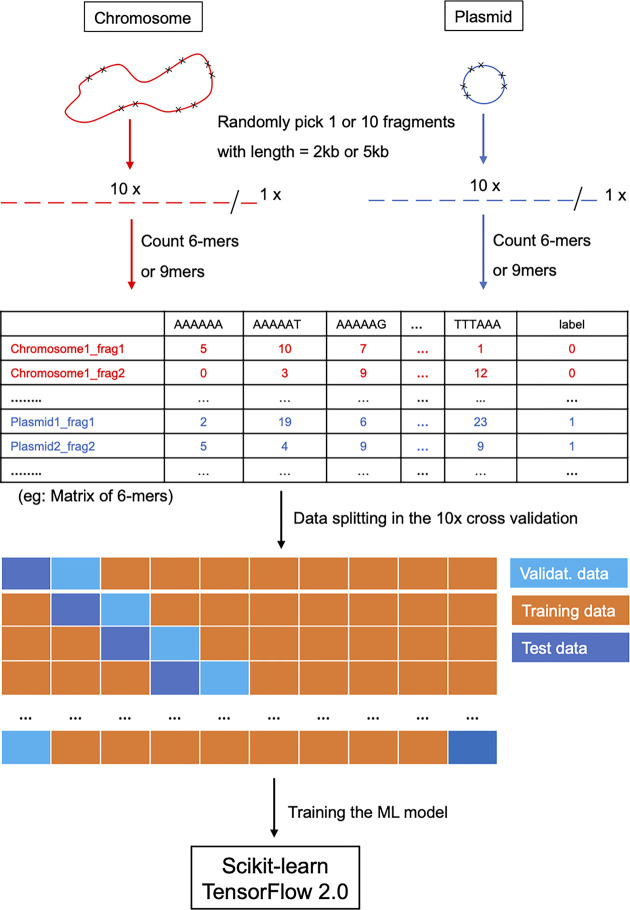
Workflow for the study.

Since the objective of this study was to develop a model that could predict whether the contigs in a draft short read assembly were from a plasmid or a chromosome, we needed a sampling strategy to accommodate various contig sequence lengths. Although previous studies have shown that longer sequences tend to produce models with higher accuracy [14,16], we wanted to have the ability to classify shorter sequences without losing the ability to classify contigs of a reasonable length. For this reason, we chose to sample 2kb and 5kb subsequences from each chromosome and plasmid contig to generate the training set. This process was replicated 10 times to obtain 10 sequences from each chromosome and plasmid (**Fig 2**).

Nucleotide k-mer counts of either 6-mers or 9-mers were then computed for each subsequence using KMC3 [23]. Shorter k-mers, tend to capture nucleotide composition patterns such as G+C content and codon usage, while longer k-mers tend to capture unique sequence strings. We chose to evaluate 6-mers and 9-mers based on their overall memory footprint on our available GPUs (**Supplemental Methods, S1 Fig in S1 File**). For each k-mer, the forward and reverse strands are considered, and only the lexicographically highest “canonical” k-mer is used in the models. In other words, to avoid double counting, the k-mer “AAAAAA” is counted rather than “TTTTTT” because either strand could have been sequenced. When the 5kb samples were used to build models, all plasmid contigs less than 5kb in length were excluded from the analysis. The dataset (exempting the holdout set) was split by 7:2:1 ratio for model training, testing, and validation, respectively.

ML models were built and tested using Python version 3.8. We computed logistic regression and random forest models using Scikit-Learn (version 0.21.3) [24], XGBoost models using XGBoost [25], and a neural network using TensorFlow 2.0 [26]. We defined a binary classification problem using the category “plasmid” as positive class and “chromosome” as the negative class. For the logistic regression model, we tuned the following hyperparameters: penalty (“l1” or “l2”), C value (0.2 to 3 with step size of 0.2) and solver (“sag” and “liblinear”). We chose the following parameters: a penalty of 12, liblinear solver, and 1,000 maximum iterations to train the final logistic regression model because it resulted in the best accuracy. For the random forest model, we tuned the following hyperparameters: n_estimators (20 to 400 with step size of 20), max_depth (default, 10 to 100 with step size of 10), and criterion (“gini” and “entropy”) to evaluate the performance of the model each iteration under these parameters and to plot the results as learning curves. In the final random forest model, the number of trees in the forest was set as 200 and the remaining parameters were set as the defaults. For the XGBoost model, a grid search was designed to determine the best parameters for eta (0 to 1 with a step size of 0.05), max_depth (5 to 50 with a step size of 2), and objective (“binary:logistic,” and “binary:hinge”). For the final XGBoost parameters, “objective” was set to “binary: logistic”, eta was set to 0.1, and the remaining parameters were set to the defaults. We tested a fully connected network from TensorFlow 2.0. The k-mer frequency matrix was scaled using StandardScaler in the sklearn package before input to the neural network models. The fully connected neural network in TensorFlow 2.0 was set to 7 layers which contained 256, 256, 128, 128, 32, 10, and 1 hidden neuron. A sigmoid activation function was chosen for the output layer while the remaining layers using a rectified linear unit activation function. Dropout layers were applied to the first 4 layers with dropout rates of 0.4, 0.4, 0.5, and 0.2, respectively. Besides the dropout layers, L2 weight regularization with value of 0.0001 was applied in the first four layers to reduce model overfitting. The model was fed by batch inputs with a batch size of 256 and trained for 200 epochs. Model hyperparameters were tuned using validation data. When we increased or decreased the number of fully connected layers and dropout layers, the model performance on the testing data set improved little. Also, few effects occurred when we adjusted the dropout rate.

### 10-fold cross-validation

Ten-fold cross validations were performed to assess the accuracy of each model and its sensitivity to the input training set. The dataset (excluding the holdout data) was divided into 10 equal parts: one part was used for testing, one part for validation, and the remaining eight parts for training. In the ten rounds, every part appears in the training, testing and validation dataset, and bias was observed by tracking the average accuracy over the test set (**Fig 2**). In each round, a receiver operating characteristic (ROC) curve was drawn and the area under the curve (AUC) was calculated using the test dataset.

### Voting classifier

For each contig in the holdout set, we chose three randomly selected sequence fragments and classified each using the tuned neural network model. We then reported the accuracy requiring only a single vote, 2 out of 3, and 3 out of 3 votes.

### Comparison to published methods

PlasFlow [17] and PlasClass [13] are two previously published ML plasmid classifiers trained using logistic regression and a neural network, respectively. Both of the models perform best for fragments greater than 10kb, but are also compatible with 5-kb fragments classification. We assessed these tools using the test data in the 10-fold cross-validation. Models were compared using the accuracy, F1 score, precision, and recall. PlasFlow predictions contain three classes: plasmid, chromosome, and unclassified. Plasflow was evaluated omitting the samples with “unclassified” predictions.

### Analysis of misclassified subsequences

The tuned neural network model was applied to 5kb subsequences drawn from the hold-out dataset containing 1,000 chromosomes and 1,000 plasmids. We sampled 5kb subsequences from the entire length of each contig. For each chromosomal contig, we sampled fragments in order using a window of 1kb as a buffer between each sampled fragment. We subsampled each plasmid into 5kb sequence fragments without using a gap since the plasmids tend to be smaller in size. Fragments with false predictions were extracted and their RAST annotations were compared [27].

### Classification of other elements

Since this model was built using bacterial chromosome and plasmid contigs, we wanted to know how the model predicted virus, phage, and insertion sequences (ISs). We download all reference viral genomes in the NCBI RefSeq database [28], all of the genomes whose assembly was labeled as phage from the European Nucleotide Archive (ENA) database [29], and all ISs in the ISfinder database [30]. The viral and phage sequences were evaluated against the 5kb model, and the IS elements were evaluated against the 2kb model since most ISs have length less than 5kb. After omitting viral and phage sequences with lengths less than 5kb, we randomly selected 10 fragments from each viral genome and one fragment from each phage genome. For ISs, we omitted sequences with length less than 2,000 bp and then randomly selected 3 fragments with length of 2kb from each IS. The selected fragments were then processed using KMC3 to get 6-mer frequencies and fed into the model to get predictions.

## Results

### Model performance using different methods

We started by building random forest and logistic regression models for classifying plasmid and chromosomal contig sequences. These were chosen because they are common ML methods that have been used for similar classification problems [31] and usually train rapidly. Since the plasmid and chromosomal contigs differ in their lengths, we randomly subsampled each contig into either 2kb or 5kb subsequences, choosing one subsequence per contig to train the models. We also tested k-mer lengths of 6 and 9 nucleotides. These k-mer lengths were chosen for evaluation because the shorter k-mers tend to capture nucleotide composition, while the longer k-mers capture specific sequence signatures. In order to provide the best performances, parameters were tuned for each model as described in the Materials and Methods.

Overall, the random forest and logistic regression models had similar accuracies in test datasets from the 10-fold cross-validation (**Table 1**). The models trained on 2kb fragments had accuracies that were approximately 5% lower than the models trained on 5kb fragments. For instance, the random forest 6-mer model had an average accuracy of 72.86% ± 2.54% using 2kb sequence fragments versus 78.13% ± 2.12% using 5kb sequence fragments (data are shown as average the accuracy ± standard deviation over 10-folds). Likewise, for logistic regression, the average 6-mer model accuracy over ten folds was 74.02% ± 2.67% for the 2kb fragment model versus 78.79% ± 1.95% for the 5kb fragment model. Higher accuracies were observed in the 9-mer models that used 5kb sequence fragments as well (**S1 Table in S1 File**). Increasing the k-mer lengths from 6 to 9 resulted in a slight improvement in the accuracy of the logistic regression model and a minor decrease in the accuracy of the random forest model (**S1 Table in S1 File**). Since the improvement in accuracy was not dramatic, and because the 6-mer models were more efficient to compute—9-mer models have 131,072 canonical k-mers compared with only 2,080 canonical k-mers for 6-mer models—we used 6-mers to train the remaining models in this study.

**Table 1:** Accuracy of ML models built for classifying plasmid and chromosome sequences using 6-mers as features*.

	Average accuracy for one randomly selected sequence fragment (%)		Average accuracy using ten randomly selected sequence fragments (%)	
Model	2kb	5kb	2kb	5kb
Random Forest	72.86 ± 2.54	78.13 ± 2.12	77.21 ± 3.14	83.98 ± 2.03
Logistic Regression	74.02 ± 2.67	78.79 ± 1.95	77.27 ± 2.46	83.17 ± 2.26
XGBoost	76.20 ± 2.21	81.56 ± 2.03	73.52 ± 1.55	79.59 ± 2.43
Neural Network	79.40 ± 3.05	85.22 ± 2.32	85.52 ± 1.87	89.38 ± 2.16

*Results are reported as the average accuracy for the test set using a 10-fold cross-validation with the standard deviation.

**Fig 3:**
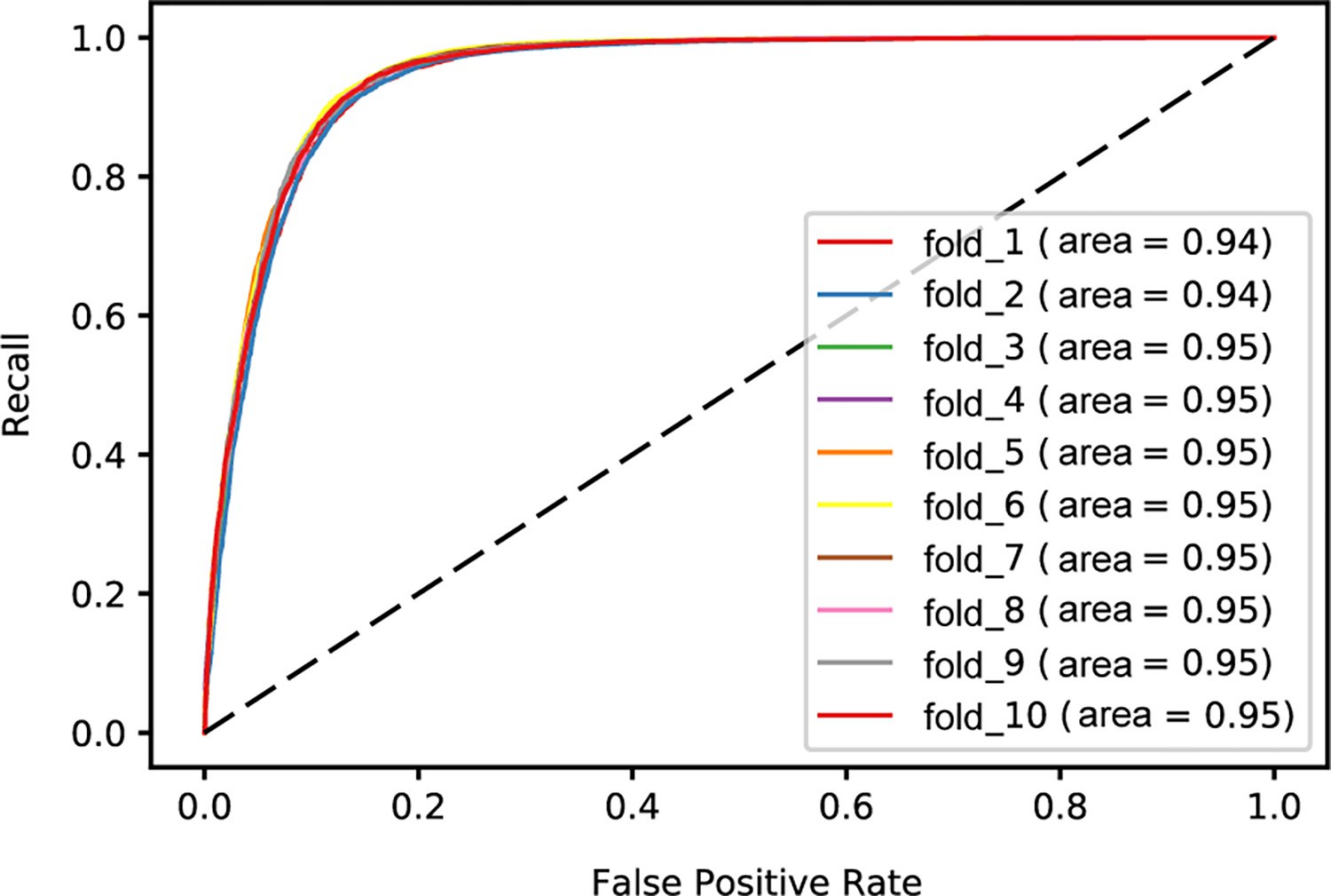
Receiver-operator characteristic curve for the 10-fold cross validated neural network model based on 6-mers and randomly selected 5-kb sequence fragments. Results for each fold are shown.

Using 6-mers as features, we also trained an XGBoost model and a fully connected neural network model on randomly selected 2kb and 5kb sequence fragments. Consistent with the logistic regression and random forest models described above, the models based on 5kb sequence fragments achieved approximately 5% higher accuracies than the models based on 2kb fragments (**Table 1**). For the XGBoost model based on 5kb sequence fragments, we observed a slight increase in accuracy of approximately 3% over the logistic regression and random forest models. Overall, the neural network model had the highest average accuracy 85.22% ± 2.32%, outperforming the other methods by approximately 3–7% (**Table 1**). For the neural network model, the area under the receiver operating characteristic curves (AUCs) for each fold of the 10-fold cross validation are consistent, ranging from 0.94 to 0.95 (**Fig 3**), indicating that this approach is stable despite the sequence variation in the training set.

**Fig 4:**
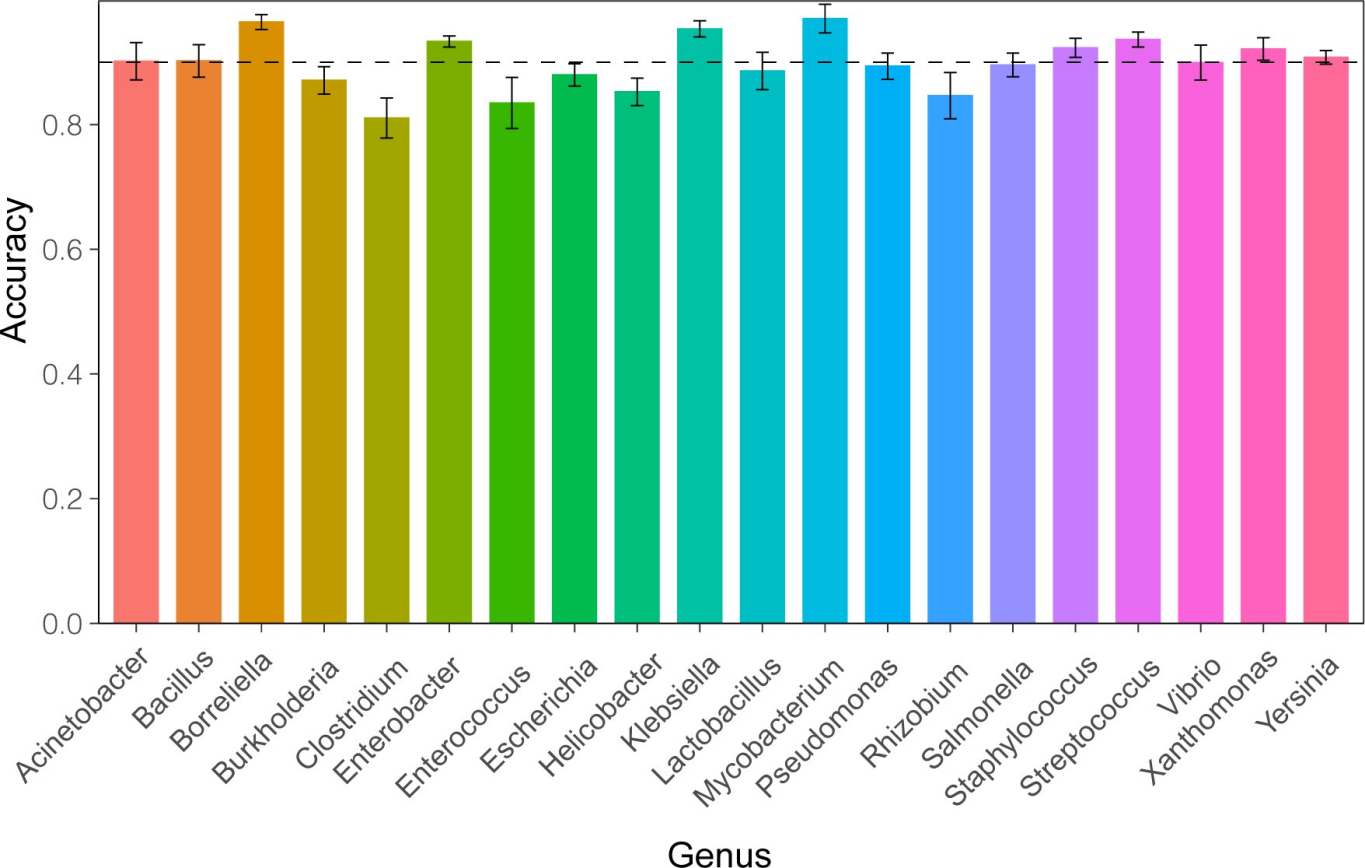
Classification accuracy for the top 20 genera in the test set. The dotted line indicates accuracy of 0.9. Error bars are the standard deviation from the result of the 10-fold cross validation.

Using the neural network model based on 5kb sequence fragments, we computed the accuracy for classifying contigs from the top 20 genera in the holdout set (**Fig 4),** using each round of the 10-fold cross-validation. All of the genera achieved accuracies over 80% and eleven had accuracies greater than 90% (**S2 Table in S1 File**). Six genera containing many pathogens of clinical significance—*Borreliella*, *Enterobacter*, *Klebsiella*, *Mycobacterium*, *Staphylococcus*, *Streptococcus—*had accuracies over 90%. The uniform accuracies across different genera indicate that this model is generalizable for the common genera.

### Sequence sampling improves accuracy

Since building a model from a single random sequence fragment per contig may not adequately represent the sequence diversity within each replicon, we built models by training 10 randomly selected 2kb or 5kb sequence fragments per contig. For all of the models except XGBoost, the average accuracy over the test set in the 10-fold cross-validation was 3–6% higher than that of individual model trained by one fragment per contig (**Table 1**). This indicates that sampling may reduce noise from any single random sequence.

Since the above results indicate that sampling improves accuracy, we developed a voting classifier using a “best out of three” strategy (**Table 2**). Three 5kb sequences were randomly drawn from each contig in the hold out set, and the results were recorded based on voting on a single sequence, voting based on 2 out of 3 sequences, and voting requiring 3 out of 3 sequences. This approach was evaluated using the random forest and neural network classifiers. Voting based on the majority (requiring 2 out of 3) improves the accuracy from 83.98% ± 2.03% to 88.11% ± 1.55% for the random forest model, and from 89.38% ± 2.16% to 92.08% ± 1.93% for the neural network model, over the single vote classifier. When 3 out of 3 votes were required, the accuracy began to diminish. Unless otherwise stated, the fully connected neural network model trained on 6-mer features and 5kb sequence fragments, using the “best out of three” strategy was chosen for further evaluation because it had the highest accuracy.

**Table 2:** Accuracy of a voting classifier for predicting chromosomal and plasmid sequences using 5kb sequence fragments from each contig and voting based on the majority*.

Votes	Random Forest (%)	Neural network (%)
Single vote	83.98 ± 2.03	89.38 ± 2.16
2 out of 3	88.11 ± 1.55	92.08 ± 1.93
3 out of 3	82.88 ± 2.63	87.20 ± 2.07

*Results are reported as the average accuracy for test set using a 10-fold cross-validation with the standard deviation.

### Analysis of false predictions

In order to understand the sequences that cause incorrect classifications, we divided each contig in the hold out set into 5kb subsequences and classified each, recording the annotations on each incorrectly classified sequence fragment. The chromosomal data set produced 525,779 incorrectly classified sequence fragments out of 6,231,904 total fragments, and the plasmid data set produced 15,478 incorrectly classified sequence fragments out of 123,084 total fragments. Among these, 226,548 (43%) of the incorrectly classified chromosomal sequence fragments and 7,619 (49%) of the incorrectly classified plasmid sequence fragments contained only annotations for "hypothetical proteins." Interestingly, among the remaining false predictions from both the plasmids and chromosomes, we found many sequence fragments that had similar annotations (**Table 3**). Subsequences encoding transporter-related functions were the most abundant source of false predictions in both the chromosome and plasmid sets. Phage-related functions, mobile elements, CRISPR-related sequences, and repeat regions were also common sources of false predictions. These functions are often indicative of horizontal gene transfer and are likely causing false predictions because of genetic exchange between the plasmids and chromosomes.

**Table 3:** The most commonly occurring annotations in sequence fragments with false predictions.

Annotations	Percentage of chromosome fragments	Percentage of plasmid fragments
Transporter-related elements*	6.9	10.8
Repeat region	6.8	3.5
Phage-related elements*	5.2	2.2
Mobile element protein*	2.8	3.6
CRISPR-related elements*	2.5	1.0
Transcriptional regulator, LysR family	0.7	1.0
Transposase	0.7	0.8
Transcriptional regulator, AcrR family	0.5	0.5
Oxidoreductase, short-chain dehydrogenase	0.4	0.7

*Annotations containing the terms “transporter”, “phage”, and “CRISPR” were grouped into Transporter-related elements, Phage-related elements, and CRISPR-related elements, respectively.

Although the purpose of this study is to evaluate ML strategies for classifying plasmid and chromosomal contigs, there are other elements such as viruses, phages, and insertion sequences (ISs) that are likely to be found as independent contigs in NGS assemblies. However, since the models are built from 6-mers, these other elements could have nucleotide composition profiles that resemble that of the plasmids or low G+C chromosomes. To see how these other elements would be classified, we collected 6,682 viral genomes from RefSeq [28], 1,959 phage genomes from ENA [29], and 5,889 ISs from the ISfinder database [30]. Each element was subdivided into 5kb sequence fragments and classified. Overall, the model assigned 41.5% of the viral sequence fragments to the plasmid class and 58.5% into the chromosomal class; 82.4% of phage sequence fragments into the chromosomal class, and 17.6% into the plasmid class; and 41.5% of the IS elements into the chromosomal class, and 58.5% into the plasmid class. In the case of viruses and IS elements, the classifications split relatively evenly over the plasmid and chromosomal classes, but the phage sequences are much more likely to be classified as being chromosomal. This may be due to a higher proportion of prophage elements in the chromosomal contigs of the training set. Overall, this suggests that developing and optimizing models that can properly capture these other sequence elements may help to improve the classification of plasmid and chromosomal contigs.

### Comparison to other methods

Two methods, PlasClass [14] and PlasFlow [16], use similar ML approaches to build classifiers to predict the plasmid or chromosomal origin of contig sequences. Using the same testing data sets in the 10-fold cross-validation, we classified 5kb sequence fragments using the PlasClass and PlasFlow models and compared their results with the accuracies of the models described above (**Table 4**). Overall, PlasFlow achieved an accuracy of 71.43% ± 4.37% over the data set, and PlasClass achieved an accuracy of 78.10% ± 3.28%. Interestingly, PlasFlow had a very high recall 90.98% ± 2.45%, indicating that it had a low false negative rate, perhaps due to it being trained with a third "unclassified" class. Although both methods perform well out of the box and have results that are consistent with the other ML methods that were evaluated, the neural network models from this study appear to have slightly better performance, although we note that the training sets and sequence lengths differed for our models and the published PlasClass and PlasFlow models. Overall, the results indicate that the neural network approach developed in this study is an effective strategy for classifying chromosomes and plasmids.

**Table 4:** Comparison of the performance of PlasFlow and PlasClass with the models built in this study for the same test dataset in the 10-fold cross validation*.

Method	Accuracy (%)	F1 score (%)	Recall (%)	Precision (%)
PlasClass	78.10 ± 3.28	82.65 ± 4.61	79.75 ± 3.26	85.77 ± 3.87
PlasFlow	71.43 ± 4.37	81.34 ± 5.66	90.98 ± 2.45	73.55 ± 5.25
Random forest	83.98 ± 2.03	84.25 ± 2.67	84.36 ± 3.06	84.14 ± 1.66
Logistic regression	83.17 ± 2.26	83.75 ± 2,71	81.65 ± 2.22	85.96 ± 2,30
XGBoost	79.59 ± 2.43	80.70 ± 1.75	80.05 ± 2.57	81.38 ± 2.11
Neural network	89.38 ± 2.16	89.67 ± 2.14	87.28 ± 2.03	87.42 ± 0.92
Neural network with best of three voting	92.08 ± 1.93	92.23 ± 2.19	91.98 ± 1.87	92.48± 3.56

*Results are reported as the average accuracy for the test set using a 10-fold cross-validation with the standard deviation.

## Discussion

Short-read next generation sequencing technologies have been the workhorse of genome sequencing for nearly twenty years, but short read assemblies are often difficult to close [32,33].

Because of this, most genomes are submitted to the public archives in draft form, which usually means that they exist in many contigs. In these data sets, unless there are long stretches of contiguity, it can be difficult to determine which contigs come from chromosomes and which come from plasmids. The problem is even worse in shotgun metagenomics, where the contigs are often from a mixture of organisms. In bacteria, genetic information usually passes vertically from parent to offspring during chromosomal replication. However, some traits including antimicrobial resistance and virulence can be encoded on plasmids, which are transmitted horizontally between recipient cells [34]. Thus, accurately identifying plasmid sequences and the genes that they encode is crucial for understanding bacterial epidemiology.

In this study, we explored various ML methods and parameters for classifying plasmid and chromosomal sequences. Although building a classifier based on two classes is straightforward, there are challenges in the experimental design. For example, since plasmids tend to be short, and chromosomes tend to be long, a simple strategy of computing k-mer counts or frequencies over the entire length of each contig tends to create imbalanced feature sets. To overcome this, we built classifiers by randomly sampling subsequences from each contig that were matched in length, either 2kb or 5kb. Unsurprisingly, we found that models built from 5kb fragments performed slightly better, which is likely due to the additional information encoded in the longer sequences. Likewise, we found that sampling each contig multiple times also improved the classification accuracy. Although using sequence fragments longer than 5kb may further improve the classification accuracy, we did not want to lose the ability to classify short contigs. It may be possible to achieve further gains in accuracy in future work by building ensembles of models that are tailored for various sequence lengths.

Our best model, which was based on a neural network using 6-mers as features and drawn from randomly sampled 5-kb subsequences from each contig, achieved an average accuracy of 92.08% ± 2.16% when we used a “best out of three” voting strategy for classifying each contig in the test set. This model achieved higher accuracies than the published PlasClass and PlasFlow models for 5-kb fragment classification [14,16], which use similar ML approaches. However, this difference is likely overstated, since retraining the PlasClass and PlasFlow models on the same data set could potentially improve their performance. Nevertheless, the neural network strategy described in this study was robust to cross validation, sequence sampling, and taxonomic diversity. Using this as a starting point, we anticipate that it may be possible to further optimize model performances with more advanced parameter tuning, sequence sampling strategies, feature sets, and hardware.

Another challenge in classifying plasmid and chromosomal sequences is the natural genetic exchange that occurs between the replicons. When we examined the incorrectly classified subsequences from each class, we found a considerable overlap in their protein annotations. These included hallmarks of horizontal gene transfer including genes encoding transporters, phage proteins, and transposases. Furthermore, when we classified viral, phage and IS elements, we found that phage have a propensity to be classified in the chromosome class, which is likely due to the presence of prophage elements integrated in the chromosomes. Future work that explores the categorization of these elements into separate classes, or that includes functional annotations, may lead to overall improvements in the models.

In conclusion, after evaluating several strategies for building ML models to classify plasmid and chromosomal sequences, we found that the neural network model that used a voting strategy yields the best accuracy relative to other methods. This method outperformed existing methods out-of-the-box, using smaller training sequence lengths. This study provides a framework for identifying and characterizing plasmid DNA in NGS data sets with important human health implications.

## Supporting information

S1 File Supporting information–contains the supporting methods, tables, and figure. (DOCX)

## Abbreviations

AUC: Area under the Receiver Operating Characteristic Curve
IS: Insertion Sequence
ML: Machine Learning
NGS: Next Generation Sequencing
PATRIC: PAThosystems Resource Integration Center
XGBoost: Extreme Gradient Boosting

## References

[1] de Been M, Lanza VF, de Toro M, Scharringa J, Dohmen W, Du Y, et al. Dissemination of cephalosporin resistance genes between Escherichia coli strains from farm animals and humans by specific plasmid lineages. PLoS genetics. 2014;10(12):e1004776. doi: 10.1371/journal.pgen.1004776 ; PubMed Central PMCID: PMC4270446. 25522320 PMC4270446

[2] Gama JA, Zilhao R, Dionisio F. Impact of plasmid interactions with the chromosome and other plasmids on the spread of antibiotic resistance. Plasmid. 2018;99:82–8. doi: 10.1016/j.plasmid.2018.09.009 . 30240700

[3] Fernandez-Lopez R, Redondo S, Garcillan-Barcia MP, de la Cruz F. Towards a taxonomy of conjugative plasmids. Current opinion in microbiology. 2017;38:106–13. doi: 10.1016/j.mib.2017.05.005 . 28586714

[4] Dong N, Sun Q, Huang Y, Shu L, Ye L, Zhang R, et al. Evolution of Carbapenem-Resistant Serotype K1 Hypervirulent Klebsiella pneumoniae by Acquisition of bla VIM-1-Bearing Plasmid. Antimicrobial agents and chemotherapy. 2019;63(9). doi: 10.1128/AAC.01056-19 ; PubMed Central PMCID: PMC6709456. 31307980 PMC6709456

[5] Kopotsa K, Osei Sekyere J, Mbelle NM. Plasmid evolution in carbapenemase-producing Enterobacteriaceae: a review. Annals of the New York Academy of Sciences. 2019;1457(1):61–91. doi: 10.1111/nyas.14223 . 31469443

[6] Oloomi M, Javadi M, Bouzari S. Presence of pathogenicity island related and plasmid encoded virulence genes in cytolethal distending toxin producing Escherichia coli isolates from diarrheal cases. International journal of applied & basic medical research. 2015;5(3):181–6. doi: 10.4103/2229-516X.165366 ; PubMed Central PMCID: PMC4606577. 26539367 PMC4606577

[7] Zhuo C, Li XQ, Zong ZY, Zhong NS. Epidemic plasmid carrying bla(CTX-M-15) in Klebsiella penumoniae in China. PloS one. 2013;8(1):e52222. doi: 10.1371/journal.pone.0052222 ; PubMed Central PMCID: PMC3558504. 23382815 PMC3558504

[8] Li LL, Norman A, Hansen LH, Sorensen SJ. Metamobilomics—expanding our knowledge on the pool of plasmid encoded traits in natural environments using high-throughput sequencing. Clinical microbiology and infection: the official publication of the European Society of Clinical Microbiology and Infectious Diseases. 2012;18 Suppl 4:5–7. doi: 10.1111/j.1469-0691.2012.03862.x . 22647039

[9] Gupta SK, Raza S, Unno T. Comparison of de-novo assembly tools for plasmid metagenome analysis. Genes & genomics. 2019;41(9):1077–83. doi: 10.1007/s13258-019-00839-1 . 31187446

[10] Johnson M, Zaretskaya I, Raytselis Y, Merezhuk Y, McGinnis S, Madden TL. NCBI BLAST: a better web interface. Nucleic Acids Res. 2008;36(Web Server issue):W5–9. Epub 2008/04/29. doi: 10.1093/nar/gkn201 ; PubMed Central PMCID: PMC2447716. 18440982 PMC2447716

[11] Carattoli A, Zankari E, Garcia-Fernandez A, Voldby Larsen M, Lund O, Villa L, et al. In silico detection and typing of plasmids using PlasmidFinder and plasmid multilocus sequence typing. Antimicrobial agents and chemotherapy. 2014;58(7):3895–903. doi: 10.1128/AAC.02412-14 ; PubMed Central PMCID: PMC4068535. 24777092 PMC4068535

[12] Galata V, Fehlmann T, Backes C, Keller A. PLSDB: a resource of complete bacterial plasmids. Nucleic Acids Res. 2019;47(D1):D195–D202. doi: 10.1093/nar/gky1050 ; PubMed Central PMCID: PMC6323999. 30380090 PMC6323999

[13] Schmartz GP, Hartung A, Hirsch P, Kern F, Fehlmann T, Müller R, et al. PLSDB: advancing a comprehensive database of bacterial plasmids. Nucleic Acids Research. 2022;50(D1):D273–D8. doi: 10.1093/nar/gkab1111 34850116 PMC8728149

[14] Pellow D, Mizrahi I, Shamir R. PlasClass improves plasmid sequence classification. PLoS Comput Biol. 2020;16(4):e1007781. Epub 2020/04/04. doi: 10.1371/journal.pcbi.1007781 ; PubMed Central PMCID: PMC7159247. 32243433 PMC7159247

[15] Arredondo-Alonso S, Rogers MRC, Braat JC, Verschuuren TD, Top J, Corander J, et al. mlplasmids: a user-friendly tool to predict plasmid- and chromosome-derived sequences for single species. Microbial genomics. 2018;4(11). doi: 10.1099/mgen.0.000224 ; PubMed Central PMCID: PMC6321875. 30383524 PMC6321875

[16] Krawczyk PS, Lipinski L, Dziembowski A. PlasFlow: predicting plasmid sequences in metagenomic data using genome signatures. Nucleic Acids Res. 2018;46(6):e35. doi: 10.1093/nar/gkx1321 ; PubMed Central PMCID: PMC5887522. 29346586 PMC5887522

[17] Pu L, Shamir R. 3CAC: improving the classification of phages and plasmids in metagenomic assemblies using assembly graphs. bioRxiv. 2022:2021.11.05.467408. doi: 10.1093/bioinformatics/btac468 36124804

[18] Fang Z, Tan J, Wu S, Li M, Xu C, Xie Z, et al. PPR-Meta: a tool for identifying phages and plasmids from metagenomic fragments using deep learning. Gigascience. 2019;8(6). Epub 2019/06/21. doi: 10.1093/gigascience/giz066 ; PubMed Central PMCID: PMC6586199. 31220250 PMC6586199

[19] Davis JJ, Wattam AR, Aziz RK, Brettin T, Butler R, Butler RM, et al. The PATRIC Bioinformatics Resource Center: expanding data and analysis capabilities. Nucleic Acids Res. 2020;48(D1):D606–D12. doi: 10.1093/nar/gkz943 ; PubMed Central PMCID: PMC7145515. 31667520 PMC7145515

[20] Olson RD, Assaf R, Brettin T, Conrad N, Cucinell C, Davis James J, et al. Introducing the Bacterial and Viral Bioinformatics Resource Center (BV-BRC): a resource combining PATRIC, IRD and ViPR. Nucleic Acids Research. 2022. doi: 10.1093/nar/gkac1003 36350631 PMC9825582

[21] Parrello B, Butler R, Chlenski P, Olson R, Overbeek J, Pusch GD, et al. A machine learning-based service for estimating quality of genomes using PATRIC. BMC bioinformatics. 2019;20(1):1–9. 31581946 10.1186/s12859-019-3068-y PMC6775668

[22] Sayers EW, Cavanaugh M, Clark K, Pruitt KD, Schoch CL, Sherry ST, et al. GenBank. Nucleic Acids Research. 2020;49(D1):D92–D6. doi: 10.1093/nar/gkz956 33196830 PMC7778897

[23] Kokot M, Dlugosz M, Deorowicz S. KMC 3: counting and manipulating k-mer statistics. Bioinformatics. 2017;33(17):2759–61. doi: 10.1093/bioinformatics/btx304 . 28472236

[24] Pedregosa F, Varoquaux G, Gramfort A, Michel V, Thirion B, Grisel O, et al. Scikit-learn: Machine learning in Python. the Journal of machine Learning research. 2011;12:2825–30.

[25] Chen T, Guestrin C, editors. Xgboost: A scalable tree boosting system. Proceedings of the 22nd acm sigkdd international conference on knowledge discovery and data mining; 2016.

[26] Abadi M, Barham P, Chen J. TensorFlow: A System for Large-Scale Machine Learning. ArXiv preprint: 1605.08695 [cs. DC](Cornell Univ. Library, Ithaca, 2016). 2022.

[27] Brettin T, Davis JJ, Disz T, Edwards RA, Gerdes S, Olsen GJ, et al. RASTtk: a modular and extensible implementation of the RAST algorithm for building custom annotation pipelines and annotating batches of genomes. Scientific reports. 2015;5:8365. doi: 10.1038/srep08365 ; PubMed Central PMCID: PMC4322359. 25666585 PMC4322359

[28] NCBI. The Reference Sequence (RefSeq) collection 2022 [cited 2019]. Available from: https://www.ncbi.nlm.nih.gov/refseq/.

[29] EMBL-EBI. European Nucleotide Archive 2019 [cited 2019 Oct 6]. Available from: https://www.ebi.ac.uk/ena.

[30] Siguier P, Perochon J, Lestrade L, Mahillon J, Chandler M. ISfinder: the reference centre for bacterial insertion sequences. Nucleic Acids Res. 2006;34(Database issue):D32–6. doi: 10.1093/nar/gkj014 ; PubMed Central PMCID: PMC1347377. 16381877 PMC1347377

[31] Couronne R, Probst P, Boulesteix AL. Random forest versus logistic regression: a large-scale benchmark experiment. BMC Bioinformatics. 2018;19(1):270. Epub 2018/07/19. doi: 10.1186/s12859-018-2264-5 ; PubMed Central PMCID: PMC6050737. 30016950 PMC6050737

[32] Ghurye JS, Cepeda-Espinoza V, Pop M. Metagenomic Assembly: Overview, Challenges and Applications. Yale J Biol Med. 2016;89(3):353–62. Epub 2016/10/05. ; PubMed Central PMCID: PMC5045144. 27698619 PMC5045144

[33] Prakash T, Taylor TD. Functional assignment of metagenomic data: challenges and applications. Brief Bioinform. 2012;13(6):711–27. Epub 2012/07/10. doi: 10.1093/bib/bbs033 ; PubMed Central PMCID: PMC3504928. 22772835 PMC3504928

[34] Rodriguez-Beltran J, DelaFuente J, Leon-Sampedro R, MacLean RC, San Millan A. Beyond horizontal gene transfer: the role of plasmids in bacterial evolution. Nat Rev Microbiol. 2021;19(6):347–59. Epub 2021/01/21. doi: 10.1038/s41579-020-00497-1 . 33469168

