# Supplementary material for: Classification of bacterial plasmid and chromosome derived sequences using machine learning
Source: PLoS One. 2022 Dec 16;17(12):e0279280. doi: 10.1371/journal.pone.0279280 (PMC9757591; doi:10.1371/journal.pone.0279280)
Supplement: S1 File — (DOCX) [file pone.0279280.s001.docx]

**Supporting Information**

**Classification of bacterial plasmid and chromosome derived sequences using machine learning**

Xiaohui Zou, Marcus Nguyen, Jamie Overbeek, Bin Cao, James J. Davis

**Supplemental Methods**

**Estimation of neural network memory usage**

Projected memory use $P$ is computed based on the projected memory use of the neural network $P_{n}$ and the projected memory use of the matrix $P_{m}$. This is computed with the following:

$$P=P_{n}+P_{m}$$

$$P_{n}=W\times64$$

$$W=c\left( k \right) \times256+256 \times256+256 \times256+256 \times128+128 \times128+128 \times128+128 \times32+32 \times10+10$$

$$P_{m}=c\left( k \right)\times21000\times64$$

$$c\left( k \right)=\{$$

$$k=6\to2080$$

$$k=7\to8192$$

$$k=8\to32896$$

$$k=9\to131072$$

$$k=10\to524800$$

$$\}$$

$P_{n}$ is based on the number of weights $W$ within the model.

$W$ is based on the model structure which contains layers of 256, 256, 128, 128, 32, and 10 nodes and the number of unique k-mers $c(k)$ for a given k-mer size $k$.

$P_{m}$ is based on the $c(k)$ and the number of samples 21000.

**Supplemental Tables**

| **S1 Table.** Accuracy of ML models for classifying plasmid and chromosome sequences using 6-mers and 9-mers as features and subsequence lengths. | | | | | | | |
| --- | --- | --- | --- | --- | --- | --- | --- |
| Sequence  Fragment | k-mer size | Chromosomes | Plasmids | Total  Features | Random Forest (%)^*^ | Logistic Regression (%)^*^ | |
| 2kb | 6-mer | 10,584 | 10,654 | 2,080 | 72.86 ± 2.54 | | 74.02 ± 2.67 |
| 2kb | 9-mer | 10,584 | 10,654 | 131,072 | 72.92 ± 3.13 | | 77.77 ± 2.94. |
| 5kb | 6-mer | 10,434 | 9,435 | 2,080 | 78.13 ± 2.12 | | 78.79 ± 1.95 |
| 5kb | 9-mer | 10,434 | 9,435 | 131,072 | 73.58 ± 2.33 | | 82.50 ± 2.76 |

^*^Average accuracy and standard deviation of test set using a 10-fold cross-validation

**S2 Table.** Classification results for the neural network model based on 5kb fragments for the top 20 genera in the holdout set (10 fragments sampled from each chromosome/plasmid).

| **Genus** | **Accuracy (%)**^*^ | **True Positives** | **True Negatives** | **False Positives** | **False Negatives** |
| --- | --- | --- | --- | --- | --- |
| Staphylococcus | 92.32 ±1.54 | 348 | 1322 | 3 | 136 |
| Escherichia | 87.98 ± 1.82 | 698 | 612 | 157 | 22 |
| Mycobacterium | 97.01 ± 2.33 | 72 | 804 | 9 | 18 |
| Klebsiella | 95.36 ± 1.32 | 732 | 49 | 30 | 8 |
| Bacillus | 90.22 ± 2.58 | 580 | 130 | 47 | 31 |
| Salmonella | 89.58 ± 1.94 | 315 | 373 | 56 | 24 |
| Acinetobacter | 90.17 ± 3.20 | 248 | 284 | 46 | 12 |
| Lactobacillus | 88.60 ± 3.18 | 384 | 90 | 34 | 27 |
| Streptococcus | 93.65 ± 1.21 | 22 | 450 | 14 | 18 |
| Rhizobium | 84.65 ± 3.75 | 363 | 1 | 59 | 7 |
| Vibrio | 89.95 ± 2.87 | 102 | 247 | 21 | 18 |
| Pseudomonas | 89.38 ± 2.11 | 107 | 238 | 29 | 12 |
| Enterococcus | 83.47 ± 4.15 | 154 | 154 | 45 | 16 |
| Borreliella | 96.46 ± 1.22 | 316 | 11 | 9 | 3 |
| Burkholderia | 87.10 ± 2.24 | 133 | 137 | 23 | 17 |
| Xanthomonas | 92.14 ± 1.83 | 169 | 42 | 18 | 0 |
| Yersinia | 90.78 ± 1.12 | 153 | 44 | 16 | 4 |
| Enterobacter | 93.33 ± 1.05 | 189 | 7 | 3 | 11 |
| Helicobacter | 85.26 ± 2.22 | 19 | 143 | 7 | 21 |
| Clostridium | 81.05 ± 3.21 | 112 | 42 | 28 | 8 |

^*^Average accuracy and standard deviation were obtained from 10-fold cross-validation; confusion matrix was obtained from the prediction result by tuned neural network model.

**Supplemental Figure**

**S1 Fig.** Estimated GPU memory usage for the neural network based on k-mer sizes 6 through10 and 1 and 2 GPUs with 32 and 64 GB of memory, respectively.
